# The Comparative Efficacy and Safety of Entecavir and Lamivudine in Patients with HBV-Associated Acute-on-Chronic Liver Failure: A Systematic Review and Meta-Analysis

**DOI:** 10.1155/2016/5802674

**Published:** 2016-04-11

**Authors:** Jiao Yang, Hang Sun, Qi Liu

**Affiliations:** Department of Infectious Diseases, Institute for Viral Hepatitis, The Second Affiliated Hospital of Chongqing Medical University, 76 Linjiang Road, Chongqing 400010, China

## Abstract

*Background.* Currently, both of entecavir and lamivudine are effective for patients with HBV-associated acute-on-chronic liver failure (ACLF). However, there is no consensus on the efficacy of entecavir versus lamivudine for patients with HBV-associated ACLF. The aim of the study was to compare the efficacy and safety of entecavir with that of lamivudine for HBV-associated ACLF patients.* Methods.* Publications on entecavir versus lamivudine in HBV-associated ACLF patients were comprehensively identified. Odds ratio and mean difference were used to measure the effect.* Results.* Ten studies, totaling 1254 patients, were eligible. No significant differences between the two drugs presented in the 1-, 2-, 3-, or 6-month survival rates. However, after 12 months of treatment, patients prescribed entecavir had a statistically higher survival rate (*p* = 0.008) and lower total bilirubin (*p* < 0.0001) and alanine aminotransferase (*p* = 0.04) levels compared to patients prescribed lamivudine. More patients achieved HBV negative levels when taking entecavir as measured at 1-, 3-, and 12-month time points and had a lower rate of HBV recurrence.* Conclusion.* While entecavir and lamivudine are both relatively safe and well tolerated, entecavir was more efficacious in terms of survival rate and clinical improvement in long-term treatment. Further prospective randomized controlled trials are needed to validate these results.

## 1. Introduction

Acute-on-chronic liver failure (ACLF), defined as a condition where acute hepatic insult occurs simultaneously with manifestation of jaundice and coagulopathy, complicated within 4 weeks by ascites and/or encephalopathy in a patient with previously diagnosed or undiagnosed chronic liver disease [1]. A major cause of ACLF in Asia is chronic hepatitis B virus (HBV) [2]. HBV-associated ACLF has an extremely poor prognosis [3]. There is no standard treatment for ACLF; rather treatment follows the paradigm of addressing the predisposing event, alleviating the inflammatory response and providing supporting care. Artificial liver support is in many cases used as a stabilizing measure for patients with ACLF. However, it is not reckoned to reduce the mortality of patients suffering from ACLF [4, 5]. Currently, liver transplantation is deemed the only really effective therapy for ACLF, but a shortage of suitable donors and the high cost of transplant surgery hinder its clinical application [6, 7]. Therefore, establishment of more effective noninvasive therapeutic strategies is urgently needed.

The mechanism of HBV-associated ACLF remains vague. Nevertheless, viral factors, host factors, and their interactions have great impact on the prognosis of ACLF [8–11]. Nucleos(t)ide analogues such as lamivudine, entecavir, telbivudine, and tenofovir disoproxil fumarate (TDF), which suppress the replication of HBV [12, 13], can improve liver function, reduce cirrhotic complications, and decrease the incidence of hepatocellular carcinoma in patients with chronic hepatitis B. More recent, encouraging studies have concluded that antiviral therapy can increase the overall survival rate and ameliorates liver function in patients with HBV-associated ACLF compared with subjects not treated with nucleos(t)ide analogues [14–16].

Entecavir is superior to lamivudine in the suppression of HBV replication with an extremely low mutation rate in both HBeAg-positive and HBeAg-negative patients [17, 18]. The theoretical cause of entecavir's success in the long-term treatment of ACLF may lie in the latter's severe reactivation of HBV. However, the clinical data on the efficacy and safety of entecavir and lamivudine contain the inconsistencies arising from the paucity of larger sample sizes, contemporary controls, and long-term research. Studies conducted by Wen et al. [19], Yuen [20], and Zhang et al. [21] have suggested entecavir's relative efficacy compared to lamivudine, while one study by Cui et al. [22] found no significant differentials between patients with HBV-associated ACLF treated with entecavir and lamivudine. Therefore, this meta-analysis was performed to explore whether a more thorough analysis of extant study data could settle the vexed question of which was safer and more effective in treating HBV-associated ACLF patients—entecavir or lamivudine?

## 2. Materials and Methods

### 2.1. Methods

The research methods follow the preferred reporting items for systematic review and meta-analysis protocols (PRISMA-P) [23].

### 2.2. Selection and Exclusion Criteria

In this meta-analysis, randomized controlled trials and cohort studies were eligible with efficacy comparison of entecavir and lamivudine for patients suffering from hepatitis B-associated ACLF.

According to the criteria of acute-on-chronic liver failure (ACLF) from both the Chinese Medical Association and Asian Pacific Association [24], a set of baseline metrics were established for judging study data on patients with hepatitis B-associated ACLF. Studies were eligible when the subjects met the following criteria:

1. The presence of serumal hepatitis B surface antigen (HBsAg) for at least 6 months.
2. HBV DNA level >10^5^ copies/mL.
3. Serum total bilirubin (TBIL) concentration >85 *μ*mol/L and plasma prothrombin activity <40% or international standard ratio (INR) ≥1.5.
4. No complications or comorbidities such as hepatic encephalopathy or abrupt and obvious increase of ascites or spontaneous bacterial peritonitis.

Studies conforming to any of the following criteria were excluded:

1. Coinfection with hepatitis A, hepatitis C, hepatitis D, hepatitis E, cytomegalovirus, or human immunodeficiency virus (HIV).
2. Other concomitant liver diseases, such as drug hepatitis, alcoholic liver disease, autoimmune hepatitis, or Wilson's disease.
3. Patients suffering from serious medical disease or tumor.
4. A previous course of any antiviral therapy during the preceding 6 months.

### 2.3. Data Collection Process

A comprehensive search was completed of the Cochrane Central Register of Controlled Trials, PubMed, Medline, Embase, China National Knowledge Infrastructure (CNKI), and the Chinese BioMedical Literature Database. In addition, reference items of the eligible studies and relevant reviews were checked for qualified studies. The following keywords were searched: “entecavir”, “lamivudine”, “nucleoside analogue”, “nucleotide analogue”, “liver failure”, “hepatic failure”, “acute on chronic liver failure”, and “chronic hepatitis B”. The search strategy used in PubMed is as follows: “(acute-on chronic liver failure [Title/Abstract]) AND (HBV) AND (lamivudine OR entecavir OR nucleoside analogues OR nucleotide analogues)”.

Two researchers (Jiao Yang and Hang Sun) independently conducted the literature retrieval, study selection, and data extraction. Differences in assessment were resolved by consensus.

### 2.4. Assessment of Study Quality

For randomized controlled trials (RCTs), the Cochrane risk of bias tool including random sequence generation, allocation concealment, blinding of participants and personnel, blinding outcome assessment, incomplete outcome data, selective reporting, and other sources of bias was used to evaluate the quality of the included studies. Newcastle-Ottawa Scale (NOS) involving the selection of cohorts, comparability of cohorts, and assessment of the outcomes was applied to assess the quality of observational cohort studies. Studies with an overall score ≥7 were defined as high-quality.

### 2.5. Efficacy Measures

The primary efficacy endpoint was overall survival rate of different time points. Secondary efficacy endpoints were recurrence rate of HBV, incidence of HBV negative, TBIL, ALT, and PTA changes as measures of hepatic improvement. The safety of entecavir and lamivudine was also assessed in the meta-analysis.

### 2.6. Data Analysis

The analysis was conducted by the use of RevMan 5.3 (Nordic Cochrane Centre, Cochrane Collaboration). A *p* value of less than 0.05 was regarded as statistically significant. Heterogeneity was assessed using the *χ* ^2^ square test and *I* ^2^ statistic. *I* ^2^ < 50% or *p* > 0.10 was considered to indicate no significant heterogeneity between studies and the fixed-effects model was employed to analyze the data. Otherwise, the random-effects model was used. Publication bias was evaluated by a funnel plot. Odds ratio (OR), mean difference (MD), and 95% confidence interval (CI) were used as effect measurements.

## 3. Results

### 3.1. Study Selection

**Figure 1:**
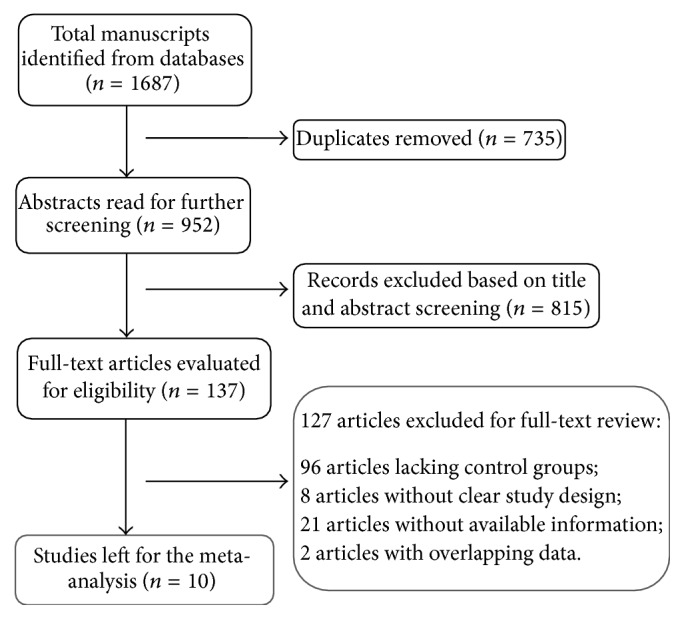
Flow diagram of literature selection process.

Of the 1687 manuscripts identified, 735 duplicates were removed. 10 studies [19, 21, 22, 25–31] were selected as eligible for the next phase of detailed analysis. 1254 patients (629 using entecavir and 625 for lamivudine) in total met the inclusion criteria for this meta-analysis (Figure 1). Of the included studies, four were randomized controlled trials, two were prospective cohort studies, and four were retrospective cohort studies. All the studies were performed in China. The dosages of entecavir and lamivudine were unified in the eligible studies. All patients included were given routine comprehensive treatment, including intensive care monitoring, nutritional supplementation, and plasma, electrolyte, and acid-base equilibrium, and prophylaxis and treatment of complications. The baseline characteristics of the eligible studies are shown in Table 1.

### 3.2. Risk of Bias in Included Studies

The overall quality of the eligible studies in this meta-analysis was suboptimal. There were four RCTs in the meta-analysis. For the RCTs, selection bias, reporting bias, and other biases were not clear, performance bias was high, and attrition bias was low (Figure 3). The quality of cohort studies was shown in Table 2. No obvious publication bias was found (Figure 4).

### 3.3. Efficacy Comparison

#### 3.3.1. Overall Survival Rates

**Figure 2:**
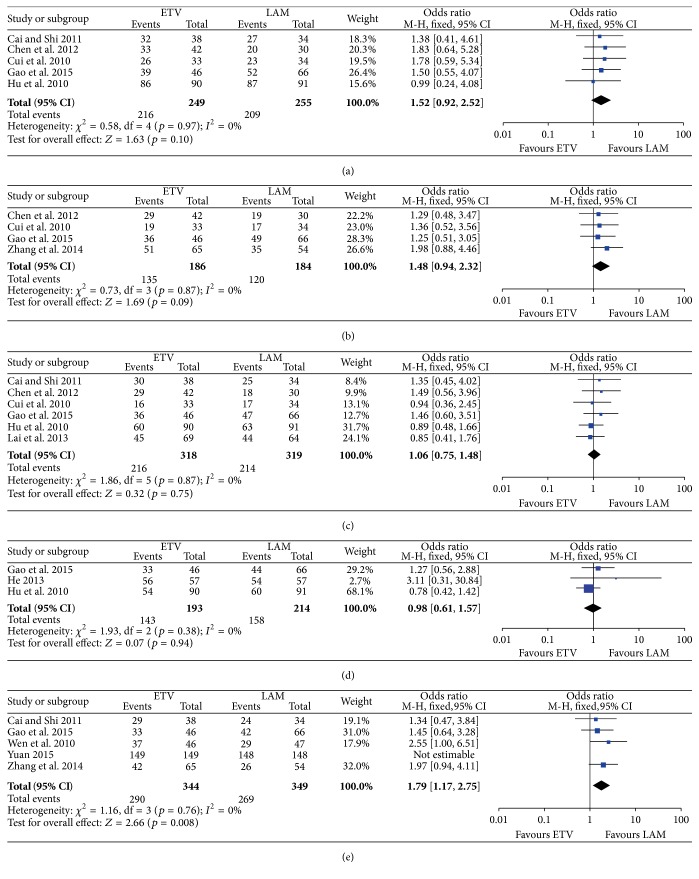
(a) Comparison of 1-month survival rates between patients taking entecavir and those taking lamivudine. (b) Comparison of 2-month survival rate between patients taking entecavir and those taking lamivudine. (c) Comparison of 3-month survival rates between patients taking entecavir and those taking lamivudine. (d) Comparison of 6-month survival rates between patients taking entecavir and those taking lamivudine. (e) Comparison of 12-month survival rates between patients taking entecavir and those taking lamivudine.

**Figure 3:**
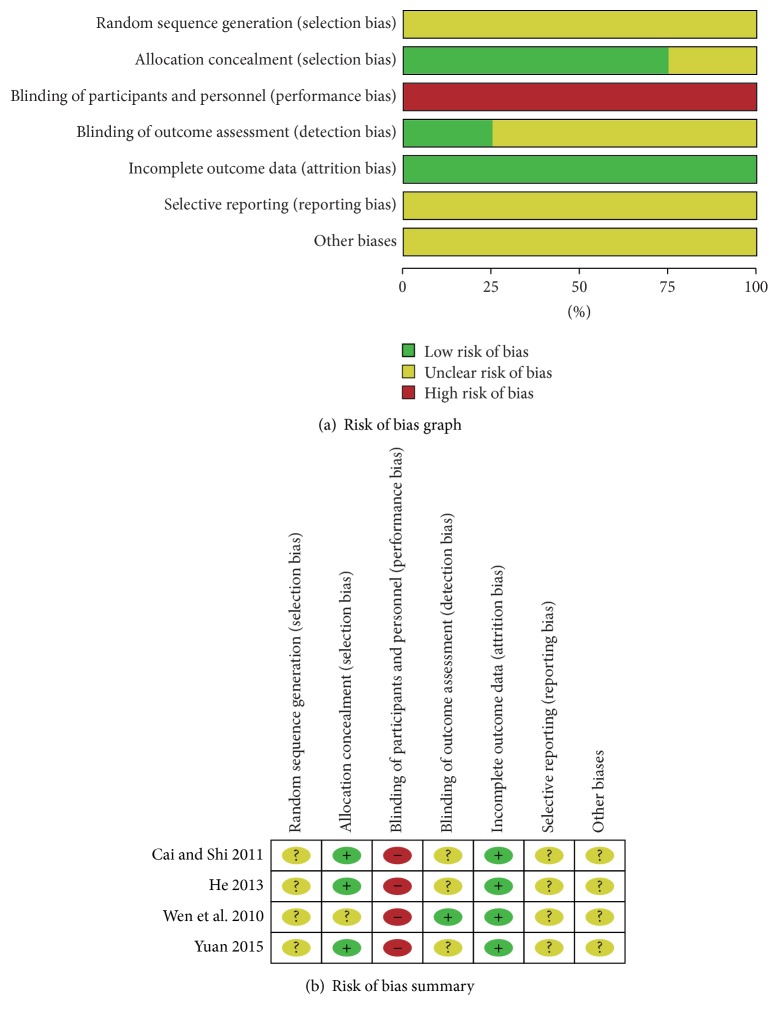
(a) Risk of bias graph: review of authors' judgments about each risk of bias item presented as percentages across all included RCTs. (b) Risk of bias graph: review of authors' judgments about each risk of bias item for each included study.

**Figure 4:**
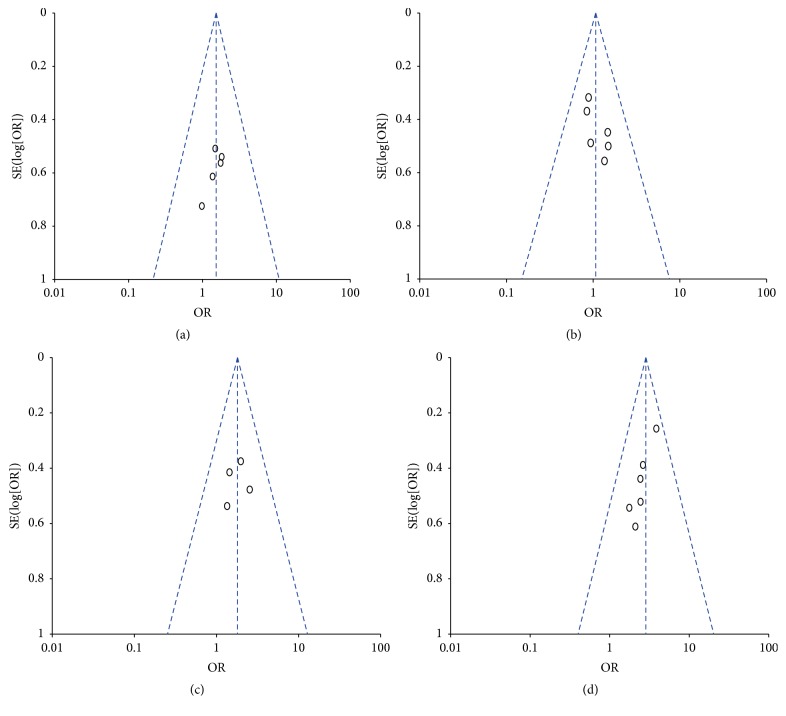
(a) Funnel plot of 1-month survival rate. (b) Funnel plot of 3-month survival rate. (c) Funnel plot of 12-month survival rate. (d) Funnel plot of the incidence of 1-month HBV negative status.

*(1) One-Month Survival Rate.* Five studies reported the details of survival rates at one month, with a total of 504 patients (249 patients using entecavir and 255 taking lamivudine). No significant heterogeneity was observed between these studies (*I* ^2^ = 0% and *p* = 0.97) and the fixed effect model was used. Comparable survival rates at this time point between patients given entecavir and those on lamivudine (86.75% versus 81.96%; OR: 1.52; 95% CI: 0.92, 2.52; *p*: 0.1) are shown in Figure 2.

*(2) Two-Month Survival Rate.* Four studies involving 186 patients using entecavir and 184 using lamivudine reported the data regarding two-month survival rates. Patients using entecavir had no significant difference in two-month survival rate compared to those on lamivudine (72.58% versus 65.22%; OR: 1.48; 95% CI: 0.94, 2.32; *p*: 0.09). The assessment of heterogeneity acquired *p* = 0.87 in Cochran's *Q* test and *I* ^2^ = 0%, meaning no variability of the included studies (Figure 2).

*(3) Three-Month Survival Rate.* Six studies provided three-month survival rate data. We included 318 patients taking entecavir and 319 using lamivudine. *I* ^2^ = 0% and *p* = 0.87 indicated no significant heterogeneity in those studies and the fixed effect model was applied. Comparative data on improvements in the three-month survival rate between patients with entecavir and those using lamivudine (67.92% versus 67.08%; OR: 1.06; 95% CI: 0.75, 1.48; *p*: 0.75) are also shown in Figure 2.

*(4) Six-Month Survival Rate.* Data regarding overall six-month survival rates were presented in three studies with 193 patients in the entecavir group and 214 in that of lamivudine. We found that entecavir was no better than lamivudine in raising the six-month survival rate for patients with chronic hepatitis B-associated acute-on-chronic liver failure (74.09% versus 73.83%; OR: 0.98; 95% CI: 0.61, 1.57; *p*: 0.94). *I* ^2^ = 0% and *p* = 0.94 showed no obvious heterogeneity among those studies (Figure 2).

*(5) Twelve-Month Survival Rate.* Five studies had information on overall twelve-month survival rates, including 344 patients taking entecavir and 349 taking lamivudine. Patients on entecavir had a higher overall survival rate than those on lamivudine (84.30% versus 77.08%; OR: 1.79; 95% CI: 1.17, 2.75; *p*: 0.008). Due to the limited heterogeneity between the eligible studies (*I* ^2^ = 0% and *p* = 0.76), the fixed effect model was used (Figure 2).

#### 3.3.2. HBV DNA Negative

*(1) One-Month HBV DNA Negative.* Six studies with 382 patients in the entecavir group and 353 in the lamivudine cohort reported the incidence of one-month HBV DNA negative changes. No significant heterogeneity was found (*I* ^2^ = 0% and *p* = 0.74). Therefore, the fixed effect model was used. Patients given entecavir presented a higher HBV DNA negative rate than subject on lamivudine at one month (65.71% versus 43.91%; OR: 2.85; 95% CI: 2.06, 3.94; *p* < 0.00001) (Table 3).

*(2) Three-Month HBV DNA Negative.* Data on the HBV DNA negative rate at three months were available in three studies. These studies included 236 patients taking entecavir and 229 taking lamivudine. More patients with entecavir achieved negative levels of HBV DNA than those with lamivudine (86.44% versus 64.63%; OR: 3.49; 95% CI: 2.20, 5.53; *p* < 0.00001). With *I* ^2^ = 0% and *p* = 0.83 the lack of significant heterogeneity led to the application of the fixed effect model was applied (Table 3).

*(3) Twelve-Month HBV DNA Negative.* Three studies comprising 215 patients on entecavir and 201 on lamivudine reported data on twelve-month HBV DNA negativity. No apparent heterogeneity was found between those studies (*I* ^2^ = 0% and *p* = 0.84). Entecavir largely enhanced rates of HBV DNA negative compared with lamivudine in hepatitis B-associated acute-on-chronic liver failure. This outcome seemed relatively stable irrespective of the duration of the antiviral therapy (Table 3).

#### 3.3.3. Recurrence of HBV

Four studies comprising 154 patients taking entecavir and 165 patients using lamivudine reported the information of recurrence of HBV. No patients in entecavir group experienced a recurrence of HBV compared to 18 in the lamivudine cohort. Entecavir treatment significantly reduced the recurrence rate of HBV in patients with HBV-associated ACLF compared to patients taking lamivudine (0% versus 10.91%; OR: 0.07; 95% CI: 0.01, 0.40; *p*: 0.003). These data are suggestive of further benefits in long-term survival (Table 3).

#### 3.3.4. TBIL Changes

Three studies reported the TBIL changes. No significant heterogeneity was found; the *I* ^2^ and *p* values of these studies at one-month, three-month, and twelve-month time points for TBIL changes were 0%, 0.42; 0%, 0.82; and 0%, 0.73, respectively. Therefore, the fixed effect model was applied. There were no significant differences between patients on entecavir and those using lamivudine in one-month and three-month TBIL changes (MD: −12.43, 95% CI: −36.43, 11.58, *p*: 0.31 for the former; MD: −1.69, 95% CI: −6.66, 3.29, *p*: 0.51 for the latter). However, TBIL reduction in the entecavir group was much more extensive than in those taking lamivudine at twelve months (MD: −8.73, 95% CI: −12.74, −4.72, *p* < 0.0001) (Table 4).

#### 3.3.5. ALT Changes

Four trials reported the level of ALT in patients receiving antiviral therapy at one month. 303 patients were taking entecavir and 286 lamivudine. *I* ^2^ = 0% and *p* = 0.64 showed no significant heterogeneity and the fixed effect model was used. Our results showed a comparable effect between entecavir and lamivudine in reducing the level of ALT at one month (MD: −4.96, 95% CI: −10.07, 0.14, *p*: 0.06) (Table 4).

Three articles included data on ALT changes at twelve months, with 215 subjects prescribed entecavir and 201 receiving lamivudine. Patients on entecavir had more reduced levels of ALT at twelve months compared to those using lamivudine (MD: −3.08, 95% CI: −6.08, −0.07, *p*: 0.04). No apparent heterogeneity was found (*I* ^2^ = 0% and *p* = 0.68) (Table 4).

#### 3.3.6. PTA Changes

Four studies, with a total of 283 subjects in their entecavir groups and 274 in lamivudine groups, reported the changes of PTA at one month. *I* ^2^ = 21% and *p* = 0.29 showed low heterogeneity and fixed effect model was applied. Patients taking entecavir presented higher rates of improvement of PTA than those with lamivudine after treatment for one month (MD: 2.12, 95% CI: 0.42, 3.82, *p*: 0.01) (Table 4).

Three studies reported the changes of PTA at three months and twelve months. No significant heterogeneity was measured between studies about PTA at three months and twelve months (*I* ^2^ = 0%, *p* = 0.76 for the former; *I* ^2^ = 0% and *p* = 0.99 for the latter). Patients with entecavir showed no significant improvement of PTA compared to those with lamivudine for three months and twelve months (MD: 1.91, 95% CI: −1.33, 5.15, *p*: 0.25; MD: 3.6, 95% CI: −1.07, 8.26, *p*: 0.13, resp.) (Table 4).

### 3.4. Safety

No studies reported serious adverse events attributable to entecavir or lamivudine, nor did they report any drug-related viral mutation. All the patients tolerated the treatment without modification of dose or early discontinuation.

### 3.5. Sensitivity Analysis

The sensitivity analysis was performed to confirm the stability of the primary analysis by excluding studies one by one. We found out that the overall survival rate, HBV DNA negative, and recurrence of HBV did not change significantly with the exclusion of any single study.

## 4. Discussion

ACLF is a serious condition with a high mortality. Nearly two-thirds of patients may die without liver transplantation [32, 33]. The mechanisms of HBV-associated ACLF are extremely intricate and complex and, as a result, not yet established. Nevertheless, one of the important mechanisms is the overactivity of immune response including the excess activity of HBcAg/HBeAg-specific T cells and involvement of B lymphocytes activity and peripheral glucocorticoid receptor expression [34].

Up to now, there has been no effective treatment for patients with HBV-associated ACLF. Therefore, it is critical to improve medical therapy for patients with HBV-associated ACLF as a key aim in extending periods of survival. Zhao et al.'s study suggested that HBV replication and mutation were the primary factor which may lead to chronic and acute liver failure [35]. As such, antiviral therapy by inhibition of HBV replication may be helpful in postponing the progression of liver failure and reducing the mortality of patients with HBV-associated ACLF. Though the efficacy of lamivudine and entecavir is controversial in the treatment of HBV-associated ACLF, recent studies had proven that both lamivudine and entecavir can decrease the mortality, improve the biochemical response, and effectively suppress the replication of HBV in patients with HBV-associated ACLF [14, 15]. Further studies have shown that a profound and rapid reduction of HBV DNA is effected by entecavir treatment but not by lamivudine. Accordingly, it appears that entecavir may be more efficacious than lamivudine in the treatment of HBV-associated ACLF.

In this meta-analysis, we made an efficacy comparison of entecavir and lamivudine in patients suffering from HBV-associated ACLF across ten eligible studies. The efficacy comparison outcomes were the overall survival rate, HBV DNA negativity, the recurrence of HBV, and the biochemical parameters (changes of TBIL, ALT, and PTA). The outcomes at different time points were different.

Though no statistically significant data was found, there was a discoverable tendency toward superiority of the entecavir therapy over the lamivudine in terms of survival rates at one, two, three, and six months. These outcomes are in accordance with Chen et al.'s study [27]. In the latter study, patients with entecavir and lamivudine had a similar accumulative survival rate during the first three months of treatment (66.7% versus 60%). However, patients with entecavir had significantly higher survival rate than those with lamivudine after treatment of twelve months. Those results suggest that entecavir outperforms lamivudine not in short-term survival but in that of the long term. Here, the short term was defined as not more than six months. The changes of TBIL and ALT were in line with the data on overall survival rates. Our results suggested a comparable efficacy in lowering the level of TBIL and ALT in subjects prescribed entecavir and those taking lamivudine, at least, in the short term. However, patients on entecavir acquired significantly lower TBIL and ALT levels after treatment for twelve months. PTA level at one month was significantly lower in the entecavir group than that in lamivudine subjects. Nevertheless, we found no significant difference in changes of PTA for patients with long-run use of entecavir and lamivudine. In general, both entecavir and lamivudine have the capacity to alleviate hepatic injury and improve liver function. But in the long run, entecavir may be superior to lamivudine in biochemical response. More patients on entecavir obtained HBV negative scores at one-, three-, and twelve-month time points than those on lamivudine, while, in addition, entecavir subjects had either very low or unmeasurable levels of HBV as compared to those on lamivudine. These results are attributed to entecavir's potency in suppressing HBV replication as well as the low level of mutations engendered by entecavir. These results are consistent with previous studies. As a whole, long-term use of entecavir could raise survival rates and improved patients' biochemical response, over and above its primary function to reduce virological replication rapidly for patients with HBV-associated ACLF.

However, there has been one perennial concern regarding entecavir administration in cases of chronic HBV-associated liver failure, which is acute lactic acidosis. A recent study reported that serious lactic acidosis occurred more often in patients with high MELD scores and multiorgan failure [36]. Patients in the eligible studies were mostly those with early- to mild-stage HBV-associated ACLF. Therefore, no serious adverse effects occurred as a result of the on occasion severe lactic acidosis in the meta-analysis.

The degree of hepatic necrosis, rather than the viral load, is the central determinant of short-term mortality in cases of HBV-associated ACLF [37, 38]. Therefore, the primary goal of antiviral therapy is as viral prevention in case of further liver transplantation and HBV reactivation. Moreover, cost may be a factor as lamivudine is cheaper than entecavir. As such, lamivudine may be a viable alternative in the first stages of treatment and restricted to short-term use. Routine switching to entecavir after liver function has improved or the adoption of the roadmap concept is reasonable treatment strategies for patients with HBV-associated ACLF [39].

Our study had a few limitations. Firstly, not all the eligible studies offered full datasets which satisfied requisite parameters. These gaps in data resulted in small sample sizes presenting different outcomes at different time points. The limited sample size itself might be deemed to weaken the validity of the conclusions. Secondly, there were only four randomized controlled trials and the quality of those RCTs was suboptimal. Therefore, higher-quality RCTs are a vital next step in validating our findings. Thirdly, all the eligible studies were conducted in China, rendering our conclusions potentially unsuitable for other populations. Fourthly, the conditions of eligible patients mainly are mild to moderate. Therefore, maybe our results are not conforming to patients with severe symptoms. Lastly, articles published in full text and published in English or Chinese were retrieved. There was a high probability of overlooking the eligible studies published in other languages or only in abstract.

## 5. Conclusion

In conclusion, short-term treatment with both entecavir and lamivudine are effective in increasing the survival rate and countering hepatic injury for patients with early-to-mild stage HBV-associated ACLF. But for long-term therapy, entecavir has more advantages than lamivudine, whether measured by survival rates or clinical improvement. In addition, entecavir and lamivudine were equally well tolerated during the treatment.

## Figures and Tables

**Table 1:** Baseline characteristics of the included studies.

Study	Region	Study design	Number of patients		HBV DNA log 10 copies/mL		HBeAg-positive		PTA (%) or INR (means ± SD)		TBIL (means ± SD) (%)		ALB (means ± SD) (%)		ALT (means ± SD) (%)		Treatment duration (months)
			ETV	LAM	ETV	LAM	ETV	LAM	ETV	LAM	ETV	LAM	ETV	LAM	ETV	LAM	
Hu et al., 2010 [25]	China	cohort	90	90	8.2 ± 1	8.2 ± 1	NA	NA	NA	NA	NA	NA	NA	NA	NA	NA	6

Gao et al., 2015 [26]	China	cohort	46	66	NA	NA	NA	NA	NA	NA	NA	NA	NA	NA	NA	NA	24

Cui et al., 2010 [22]	China	cohort	33	34	5.9 ± 1.5	5.9 ± 1.5	10	13	2.27 ± 0.55	2.61 ± 1.03	20.10 ± 11.24	19.91 ± 8.56	33.36 ± 4.43	31.64 ± 5.32	364 (47–2861)	226.5 (22–2314)	3

Chen et al., 2012 [27]	China	cohort	42	30	7.04 ± 1.6	7.25 ± 0.89	NA	NA	34.88 ± 12.27	32.18 ± 11.44	326.29 ± 201.35	332.65 ± 182.65	31.45 ± 5.79	29.59 ± 5.63	324.19 ± 310.04	287.61 ± 261.50	3

Lai et al., 2013 [28]	China	cohort	93	89	NA	NA	NA	NA	NA	NA	NA	NA	NA	NA	Na	NA	3

Zhang et al., 2014 [21]	China	cohort	65	54	7 ± 1.4	7.2 ± 1.6	21	23	24.7 ± 6.0	25.1 ± 5.7	331.6 ± 74.8	320.1 ± 82.4	28.7 ± 6.9	29.4 ± 5.3	352.5 ± 77.2	345.2 ± 89.5	13

Wen et al., 2010 [19]	China	RCT	46	47	NA	NA	NA	NA	29.8 ± 8.5	30.6 ± 9.1	375.2 ± 200.3	389.4 ± 198.1	31.4 ± 4.6	32.7 ± 3.9	402.5 ± 292.7	395.8 ± 297.4	12

Cai and Shi, 2011 [29]	China	RCT	38	34	NA	NA	NA	NA	34.6 ± 8.1	34.8 ± 9.6	256.5 ± 137.4	257.6 ± 135.9	31.4 ± 4.6	32.8 ± 4.5	395.4 ± 235.3	387.1 ± 245.4	12

He, 2013 [30]	China	RCT	57	57	6.2 ± 2.1	6.1 ± 1.9	NA	NA	30 ± 8	30 ± 8	339 ± 135	342 ± 148	31 ± 6	31 ± 6	399 ± 245	404 ± 237	6

Yuan, 2015 [31]	China	RCT	149	148	NA	NA	NA	NA	33.89 ± 7.78	33.96 ± 7.80	260.41 ± 140.11	260.38 ± 140.10	31.88 ± 6.11	31.89 ± 6.10	396.51 ± 240.12	397.00 ± 240.22	12

ETV: entecavir; LAM: lamivudine; NA: not available; PTA: prothrombin activity; INR: international standard ratio; TBIL: total bilirubin; ALB: albumin; ALT: alanine aminotransferase; RCT: randomized controlled studies.

**Table 2:** Quality assessment of the eligible observational cohort studies.

Studies included	Selection				Comparability		Outcome			Scores
	1	2	3	4	5	6	7	8	9	
Hu et al., 2010 [25]	**√**	**√**	**√**	**√**	**√**	**√**		**√**		**7**
Gao et al., 2015 [26]	**√**	**√**	**√**	**√**	**√**	**√**		**√**	**√**	**8**
Cui et al., 2010 [22]	**√**	**√**	**√**	**√**	**√**			**√**		**6**
Chen et al., 2012 [27]	**√**	**√**	**√**		**√**			**√**		**5**
Lai et al., 2013 [28]	**√**	**√**	**√**		**√**	**√**		**√**		**6**
Zhang et al., 2014 [21]	**√**	**√**	**√**		**√**			**√**	**√**	**6**

For cohort studies, 1 indicates exposed cohort truly representative; 2 nonexposed cohort drawn from the same community; 3 ascertainment of exposure; 4 outcome of interest not present at start; 5 cohorts comparable based on the most important factors; 6 cohorts comparable on other factors; 7 quality of outcome assessment; 8 follow-up long enough for outcomes to occur; and 9 adequacy of follow-up of cohorts.

**Table 3:** Efficacy comparison of entecavir and lamivudine for dichotomous outcomes.

Outcome of interest	Number of studies	Entecavir		Lamivudine		Effect estimate		Heterogeneity	
		Sample size	Events	Sample size	Events	OR (95% CI)	*p* value	*I* ^2^ (%)	*p* value
Overall survival									
1 month	5	249	216	255	209	1.52 (0.92, 2.52)	0.1	0	0.97
2 months	4	186	135	184	120	1.48 (0.94, 2.32)	0.09	0	0.87
3 months	6	318	216	319	214	1.06 (0.75, 1.48)	0.75	0	0.87
6 months	3	193	143	214	158	0.98 (0.61, 1.57)	0.94	0	0.38
12 months	5	344	290	349	269	1.79 (1.17, 2.75)	0.008	0	0.76
HBV DNA negative									
1 months	6	382	251	353	155	2.85 (2.06, 3.94)	<0.00001	0	0.74
3 months	3	236	204	229	148	3.49 (2.20, 5.53)	<0.00001	0	0.83
12 months	3	215	208	201	156	8.61 (3.79, 19.59)	<0.00001	0	0.84
Recurrence of HBV	4	154	0	165	18	0.07 (0.01, 0.40)	0.003	0	0.93

OR: odds ratio; CI: confidence interval.

**Table 4:** Efficacy comparison of entecavir and lamivudine for continuous outcomes.

Outcome of interest	Number of studies	Sample size		Effect estimate		Heterogeneity	
		Entecavir	Lamivudine	MD (95% CI)	*p* value	*I* ^2^ (%)	*p* value
TBIL level							
1 month	3	134	126	−12.43 (−36.43, 11.58)	0.31	0	0.42
3 months	3	236	229	−1.69 (−6.66, 3.29)	0.51	0	0.82
12 months	3	215	201	−8.73 (−12.74, −4.72)	<0.0001	0	0.73
ALT level							
1 month	4	303	286	−4.96 (−10.07, 0.14)	0.06	0	0.64
12 months	3	215	201	−3.08 (−6.08, −0.07)	0.04	0	0.68
PTA level							
1 month	4	283	274	2.12 (0.42, 3.82)	0.01	21%	0.29
3 months	3	236	229	1.91 (−1.33, 5.15)	0.25	0	0.76
12 months	3	215	201	3.6 (−1.07, 8.26)	0.13	0	0.99

TBIL: total bilirubin; ALT: alanine aminotransferase; PTA: prothrombin activity; MD: mean difference; CI: confidence interval.
